# Comparing adaptive and fixed bandwidth-based kernel density estimates in spatial cancer epidemiology

**DOI:** 10.1186/s12942-015-0005-9

**Published:** 2015-03-31

**Authors:** Dorothea Lemke, Volkmar Mattauch, Oliver Heidinger, Edzer Pebesma, Hans-Werner Hense

**Affiliations:** Institute of Epidemiology and Social Medicine, Medical Faculty, Westfälische Wilhelms-Universität Münster, Münster, Germany; Institute for Geoinformatics, Geosciences Faculty, Westfälische Wilhelms-Universität Münster, Münster, Germany; Epidemiological Cancer Registry North Rhine-Westphalia, Münster, Germany

## Abstract

**Background:**

Monitoring spatial disease risk (e.g. identifying risk areas) is of great relevance in public health research, especially in cancer epidemiology. A common strategy uses case-control studies and estimates a spatial relative risk function (sRRF) via kernel density estimation (KDE). This study was set up to evaluate the sRRF estimation methods, comparing fixed with adaptive bandwidth-based KDE, and how they were able to detect ‘risk areas’ with case data from a population-based cancer registry.

**Methods:**

The sRRF were estimated within a defined area, using locational information on incident cancer cases and on a spatial sample of controls, drawn from a high-resolution population grid recognized as underestimating the resident population in urban centers. The spatial extensions of these areas with underestimated resident population were quantified with population reference data and used in this study as ‘true risk areas’. Sensitivity and specificity analyses were conducted by spatial overlay of the ‘true risk areas’ and the significant (α=.05) p-contour lines obtained from the sRRF.

**Results:**

We observed that the fixed bandwidth-based sRRF was distinguished by a conservative behavior in identifying these urban ‘risk areas’, that is, a reduced sensitivity but increased specificity due to oversmoothing as compared to the adaptive risk estimator. In contrast, the latter appeared more competitive through variance stabilization, resulting in a higher sensitivity, while the specificity was equal as compared to the fixed risk estimator. Halving the originally determined bandwidths led to a simultaneous improvement of sensitivity and specificity of the adaptive sRRF, while the specificity was reduced for the fixed estimator.

**Conclusion:**

The fixed risk estimator contrasts with an oversmoothing tendency in urban areas, while overestimating the risk in rural areas. The use of an adaptive bandwidth regime attenuated this pattern, but led in general to a higher false positive rate, because, in our study design, the majority of true risk areas were located in urban areas. However, there is a strong need for further optimizing the bandwidth selection methods, especially for the adaptive sRRF.

**Electronic supplementary material:**

The online version of this article (doi:10.1186/s12942-015-0005-9) contains supplementary material, which is available to authorized users.

## Introduction

Investigating the spatial distribution of diseases has a long tradition in epidemiology: it provides important insights into the spatial processes of disease development and disease etiologies. Thus, the demand for spatial disease surveillance programs is rising, both for chronic diseases such as cancer or multiple sclerosis, and for infectious diseases, as the recently implemented project for contagious disease surveillance (http://www.healthmap.org) indicates. A common methodological strategy in monitoring spatial disease risks is to use regional count data as a reference. In most countries, administrative population counts are freely available only at a spatial level that does not provide sufficiently high resolutions for the demands and applications of a small-area disease surveillance [1]. Alternatively, case–control designs have been developed and employed for research purposes. This design has certain advantages through its ability to distinctly identify localized risk elevations which tend to be blurred in aggregated data [1-4]. A common risk measure used in a spatial case–control design is the spatial relative risk function (sRRF) computed via kernel density estimation (KDE), usually employing odds ratios [5-10]. The estimation of the sRRF has successfully been applied in many different areas of spatial epidemiology [1,11-14]. However, the critical point with any KDE is the optimal choice of the smoothing parameter, i.e., the bandwidth that is applied: if the bandwidth is too large the data will be over-smoothed and local extremes might be missed; on the other hand, if the bandwidth is chosen too small the data will be under-smoothed and the estimator appears “hairy” (with many modes). Many statistical methods have been suggested in order to find the optimal bandwidth and typical bandwidth selection methods are Likelihood Cross Validation (LCV [15,16], Least Squares Cross Validation (LSCV [17,18], Biased Cross Validation (BCV [19], Smoothed Cross Validation(SCV [20], and the direct-plug-in method [21]. Detailed descriptions of these bandwidth selection methods have been provided [22-25]. Nevertheless, and despite progress in finding the optimal bandwidth, KDE may still reflect the true density insufficiently when the data are very skewed or show a multimodal structure [26].

Commonly, sRRF are computed using a fixed bandwidth, that is, with the same amount of smoothing being applied for cases and controls over the entire study area. However, considering that the source population in reality is not homogeneously distributed across space, it appears more reasonable to assign less smoothing to areas with a high population density and more smoothing to regions with a lower population density. This is known as the adaptive bandwidth approach [27]. Davies and Hazelton [28] found theoretical and practical advantages in the application of the adaptive bandwidth to clinical data and implemented it in the R package *sparr* [29].

In this study we evaluate different bandwidth selection methods and the resulting fixed and adaptive bandwidth-based risk estimator using the R package *sparr*. To date, the presumed advantages of certain approaches have been evaluated only in simulation studies using theoretical data and scenarios [28]. However, many disease surveillance systems, such as cancer registries, are faced with a demand for a regional monitoring of small-area disease risk based on individual cases of disease. To address the general lack of comparison of these two methods under real-world conditions, we create a risk surface employing methods that use real-world data and that artificially produce urban ‘risk areas’. Finally, we assess the performance of the sRRF, using fixed and adaptive bandwidth-based kernel density estimation, to detect these urban ‘risk areas’.

## Methods

### Incident cancer cases

**Figure 1:**
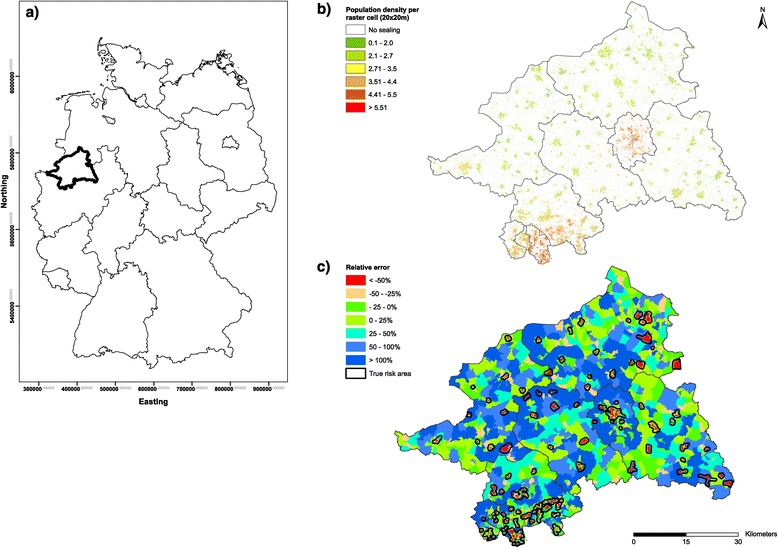
**Overview of the used data sources. (a)**: Location of the study area in Germany. **(b)**: Disaggregated, high resolution population grid using the EEA Fast Track Service Precursor on Land Monitoring dataset. **(c)**: Relative errors of the disaggregated population estimates using reference data at census tract level (N = 1,983).

Data on individual incident cancer cases were obtained from the epidemiologic cancer registry of North-Rhine Westphalia [30]. The records for all 199,280 cancer cases arising between 1986 and 2005 in the Regierungsbezirk Münster (an administrative district in the Northwest of Germany with a total population 2.7 million) (Figure 1a) were geo-coded. The geo-coding was performed by the NRW state office for information and technology [31].

For this study, we restricted the study period from 1994 to 1998 and the study population to the age group 40 to 79 years. This age range was chosen because the incidence of cancer is too low for regional surveillance programs at younger ages and of only limited public health relevance above that age. The time period was chosen because of a high geo-coding rate (95.2%) for this region and a high completeness of cancer case notification (>90%). This study was restricted to incident cases of lung cancer (ICD 11: C34) for both sexes, breast cancer (ICD 11: C50) in females only, prostate cancer (ICD 11: C61), and all cancer for both sexes. The sample sizes are given in Table 1.

**Table 1:** **Estimated bandwidths [m] and nearest neighborhood ratios (NN-ratio)**

	**N**	**Fixed bandwidth**	**Adaptive bandwidth**			**Nearest Neighbour ratio**	
**Cancer type**	**N**_**f**_ **= N**_**g**_	**OS h(pooled)**	**LSCV h(f)**	**LSCV h(g)**	**OS’ h(pooled)**	**f(NN_ratio)**	**g(NN_ratio)**
Lung cancer (male)	4826	6802.04	918.08	637.53	7635.04	0.36	0.47
Lung cancer (female)	1189	8753.08	1008.07	873.61	9825.00	0.41	0.55
Breast cancer (female)	5628	6881.42	840.50	566.05	7724.13	0.35	0.46
Prostate cancer	2926	7839.94	713.21	713.50	8800.03	0.40	0.39
Cancer all (male)	20213	5438.46	522.82	530.88	6104.47	0.32	0.42
Cancer all (female)	18019	5642.10	564.36	529.21	6333.01	0.30	0.42

‘Pooled’ refers to entire sample, ‘f’ refers to cases and ‘g’ refers to control sample.

### Sampling of spatial controls and risk areas

In order to create a surface of ‘risk areas’, a high-resolution (HR) population grid (grid size 20 meters) (Figure 1b) was constructed for the Regierungsbezirk Münster (RB-MS). We used ancillary land cover data that were based on remote sensing information about the degree of sealing (0 - 100%) [32] and population at community level (age group 40–79 years; averaged for the period 1994–1998). This approach is known to produce systematic errors, that is, population sizes in urban areas were grossly underestimated while population sizes in rural areas were overestimated, mostly due to a lack of knowledge of the height of buildings or floor area ratio [33-35].

To quantify this systematic error, the resulting HR population grid was evaluated against population reference at census tract level which provided a higher spatial resolution (N = 1,983) than the population source data at community level. The deviations of the population grid from the reference data were calculated as relative errors (RE_b_) for each grid cell (Figure 1c) by:

1 $$ R{E}_b=\left(\frac{{\widehat{y}}_b-{y}_b}{y_b}\right)100, $$

with *ŷ*_b_ the estimated number of inhabitants and *y*_b_ the true number of inhabitants in census tract *b*.

In this study, those census tracts where the relative error for the population density estimate was below −50%, denoting the third quartile (0.75) of the error distribution, were subsequently assigned as ‘risk areas’. These ‘risk areas’ were spatially buffered with a 500 m radius in order to obtain more smoothed borders of the risk areas.

Samples of spatial controls (which represent the spatial distribution of the disease-free population) were proportionally drawn from this biased HR population grid as spatial point coordinates in a 1:1 sampling design using the function “genrandompts” in the freely available Geospatial Modeling Environment (GME) (version 0.7.2.0) [36].

### Predicting spatial relative risk function (sRRF) via adaptive and fixed kernel density estimation

For arbitrary point coordinates x, usually taken over a regular grid, the sRRF is defined as the (log-transformed) ratio $\widehat{\rho}(x)$ of bivariate KDEs from cases $\widehat{f}(x)$ and controls *ĝ*(*x*), conditional on their respective sample sizes [5,6].

2 $$ \widehat{\rho}(x)=\left(\frac{\widehat{f}(x)}{\widehat{g}(x)}\right) $$

The bivariate KDE for cases $\widehat{f}(x)$ and controls *ĝ*(*x*) at location x can be written as [29]:

3 $$ \widehat{f}(x)=\frac{1}{n}{\displaystyle \sum_{i=1}^n}{h}_i^{-2}K\left(\frac{x - {X}_i}{h_i}\right), $$

where K is the Gaussian kernel function with a radially symmetrical probability density function, x_1_, …, x_n_ the bivariate coordinates of the case and, respectively, control locations, and h_i_ the bandwidth for the i-th observation.

For the estimation of the *fixed* KDE-based sRRF a constant degree of smoothing (h_i_ = h_fix_) is applied to all observations. A constant bandwidth was applied to the cases and the controls using the oversmoothing principle (OS) described by Terrell [37] in order to benefit from a first-order bias cancellation in areas where *f* ≅ *g* [9]. In contrast, for the *adaptive* KDE the bandwidth is inversely related to the population density as defined by Davies and Hazelton [28]:

4 $$ {h}_i=\frac{h_0}{f{\left({X}_i\right)}^{1/2}\gamma }, $$

where h_0_ refers to the global bandwidth, which is scaled by the product of the inverse square-root of the pilot density (f(X_i_)^-0.5^) and the geometric mean (γ) of this term. Thus, two bandwidths must be selected: a pilot and a global bandwidth. The pilot bandwidth is needed to replace the unknown density with an estimate, which is itself a fixed-bandwidth kernel estimate. For estimating pilot densities, a fixed bandwidth was applied using the least-squares cross validation (LSCV) approach [38]. The pilot bandwidths were calculated separately for the case and control data in order to assist in preserving the spatial heterogeneity in pilot densities [28]. For the global bandwidth, which is adjusted by the pilot density estimate, a common value for both data was used, implementing again the OS principle [37]. The relative risk function is then expressed as the ratio of the case and control densities f and g, respectively [5,6]. In order to symmetrize the treatment of the two density estimates and to stabilize the numerical results, the ratio was log-transformed [5,6,28,29]. To avoid boundary effects, the correction method of Diggle [39] was used for the fixed sRRF and for correcting the adaptive sRRF we applied the methods described in [40].

Different bandwidth sizes were applied for the adaptive and fixed risk estimator in sensitivity analyses (see Additional files 1, 2 and 3). For the fixed sRRF, two scenarios were implemented, based on halving (S1_fix) and doubling (S4_fix) the OS bandwidth. For the adaptive sRRF, four scenarios were set up: in the first scenario (S1_ad) the pilot (LSCV for cases and controls) and the global (OS) bandwidth were halved, and in the fourth scenario (S4_ad) both bandwidth were doubled. For the second scenario (S2_ad) the pilot bandwidth was halved and the global bandwidth was doubled and vice versa for the third scenario (S3_ad).

### Statistical analyses

To extract significant areas of risk elevations (H_0_: *r(x)* = 0; H_1_: *r(x)* > 0), based on the test statistic r(x), tolerance contours (at a significance level of α = 0.05) were calculated using the z-statistic-based asymptotic normality test [28]. This approach approximates the variances of the fixed and adaptive sRRF to construct test statistic z(x) at each location x, using the asymptotic theory of the kernel estimator z(x) ~ N(0,1), where N(0,1) denotes the standard normal distribution [28,41].

The spatial relative risk functions with the p-value contours were estimated using the R package *sparr* version 0.3-3 from [29]. This package is freely available on the Comprehensive R Archive Network [42]. All maps were projected in the ETRS89/ UTM 32N coordinate reference system [43].

### Overlay analyses of ‘risk areas’

Based on the spatial overlay of the ‘true risk areas’ with the areas of significant tolerance contours (α-level =0.05), the area portions of correctly detected risk regions (True Positives, TP) were calculated. Also, the area portions that were falsely detected (False Positives, FP) and of non-detected risk areas (False Negatives, FN) as well as that of correctly assigned non-risk areas (True Negatives, TN) were determined. We calculated for each cancer type and bandwidth method the sensitivity as Se = TP/(TP + FN), i.e. the proportion of correctly detected ‘risk areas’, and the specificity as Sp = TN/(FP + TN), i.e. the proportion of correctly assigned non-risk areas. Furthermore, the likelihood ratio was calculated as LR+ = Se/(1-Sp) = (TP/(TP + FP))/(FP/(FP + TN)), indicating how many times more likely a significant p-contour was in a risk area as compared to a non-risk areas.

### Ethics statement

The data used in this study were stored by the Epidemiological Cancer Registry North Rhine-Westphalia with doubly encrypted personal identifiers. The procedure is precisely defined by state legislation and does not require personal consent of cancer patients. Data were transferred to the investigators in an anonymized form. Use of anonymized data for research purposes does not require a vote by ethics committee or an institutional review board.

## Results

**Figure 2:**
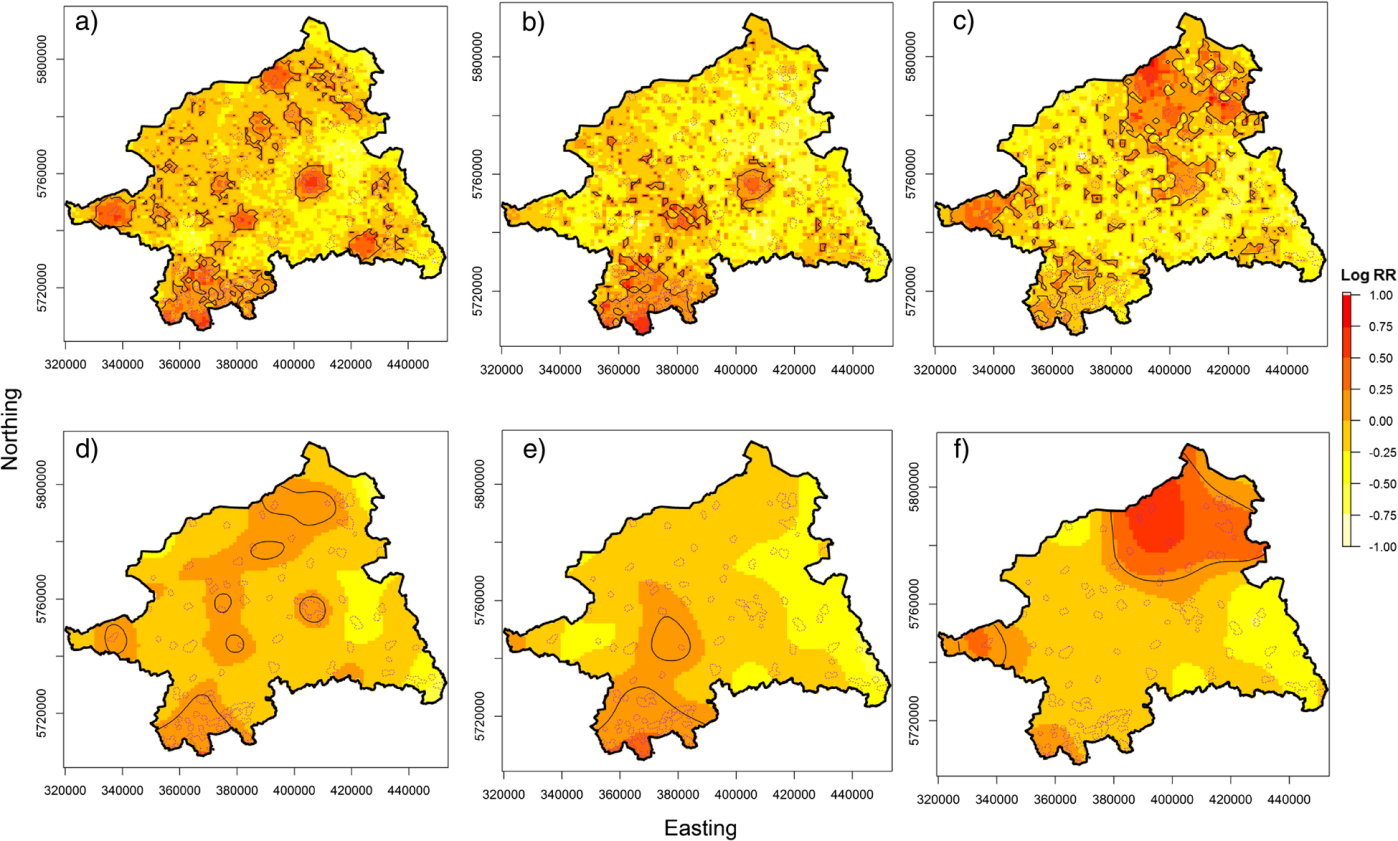
**Estimation of the spatial relative risk function for different cancer types in males.** Use of adaptive **(a** - **c)** and fixed bandwidth **(d** – **f)**; **a**. & **d**. refer to cancer all, **b**. & **e**. refer to lung cancer, **c**. & **f**. refer to prostate cancer. The 5% significant tolerance contours are overlaid as solid black lines and the true risk areas as dotted purple lines.

**Figure 3:**
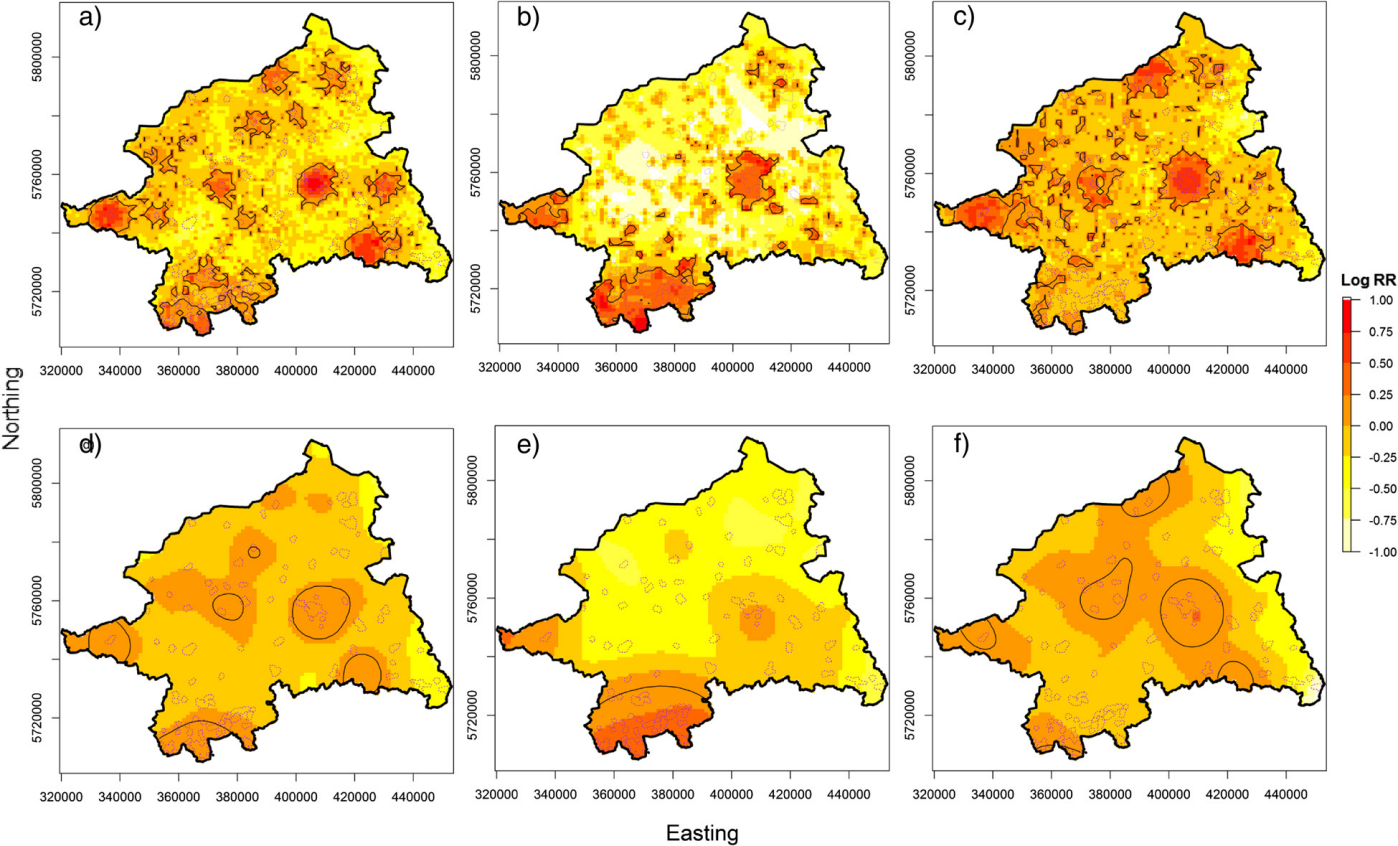
**Estimation of the spatial relative risk function for different cancer types in females.** Use of adaptive **(a** - **c)** and fixed bandwidth **(d** – **f)**; **a**. & **d**. refer to cancer all, **b**. & **e**. refer to lung cancer, **c**. & **f**. refer to breast cancer. The 5% significant tolerance contours are overlaid as solid black lines and the true risk areas as dotted purple lines.

Individual cancer cases and controls sampled (with bias) from the HR population grid were obtained to estimate the sRRF using automatically determined (data-driven) optimal bandwidths. Table 1 compares the results of the bandwidth selection methods. The table includes additionally the nearest neighborhood ratio (NN_ratio) which is the quotient of the averaged distances of each point to its closest neighbor and the mean distance of a random distribution, calculated separately for the case and the control distribution. Values < 1 indicate a clustered and values > 1 a dispersed pattern as compared to the completely spatial random distribution. The NN-ratio was calculated using the function ‘nndist’ from the *spatstat* package [44] (a package for analyzing spatial point pattern data). From Table 1 it can be seen that both selectors, the OS and the LSCV, showed narrowing bandwidths with increasing sample sizes. Of note, the OS-based bandwidths were eight to ten times larger than those based on LSCV. Moreover, the global bandwidth, OS’h(pooled), was generally larger than the bandwidths achieved with OS h(pooled). The LSCV-based bandwidths were calculated separately for cases and controls and they were mostly wider for the cases than for the controls, except for prostate cancers and all cancers in females. The NN-ratios indicated consistently that the spatial case patterns were more clustered than the control patterns, except for the prostate cancer type. Figure 2 (men) and Figure 3 (women) show the estimated values of the sRRF based on adaptive and fixed bandwidths. Significant contour lines derived from asymptotic normality tests and the outlines of the true risk areas were superimposed upon the sRRF maps. The adaptive risk estimator produced a more heterogeneous risk surface with larger differences in the logRR and significant areas that were more frequent, generally larger and more heterogeneous in size than those obtained with the fixed bandwidth approach. In the latter, areas with significantly elevated risk were confined by more smoothed contour lines.

Interestingly, the sRRF for different cancer types showed contrasting spatial patterns. Thus, the significant risk areas for ‘all cancers’ were mainly located around urban centers while the risk areas for ‘lung cancer’ were mainly located in the southern study area which covers the northern Ruhr district, the largest urban-industrial agglomeration in Germany. By contrast, sRFF analyses for ‘prostate cancer’ and ‘breast cancer’ revealed few or no risk areas in the south. ‘Prostate cancers’ produced a large pronounced risk area in the north of the study region, while the significant risk areas for ‘breast cancer’ clustered around urban areas.

Overlay analyses compared the agreement between the significant tolerance contours and the ‘true risk areas’ are given in Table 2. When regarding ‘all cancers’, the adaptive risk estimator showed a slightly better sensitivity than the fixed sRRF but this was achieved at the cost of a reduced specificity and a lower LR+. For ‘lung cancer’ the sensitivity was also slightly higher with the adaptive approach, but specificity and LR+ were high for both approaches. Although the sensitivities for both ‘prostate cancer’ and ‘breast cancer’ were rather low with the adaptive and the fixed approach, the sRRF for ‘prostate cancer’ had a slightly higher sensitivity with equal specificity. The adaptive sRRF for ‘breast cancer’ in women showed a sensitivity similar to the fixed approach but a lower specificity.

**Table 2:** **Summary of the spatial overlay analysis of the ‘risk areas’ with the significant tolerance contours (**α **= .05): Sensitivity, specificity, and the positive likelihood ratio (LR+) are presented as area ratios**

**Bandwidth**	**Cancer type**	**Sensitivity**	**Specificity**	**LR+**
Adaptive	Lung cancer (male)	0.33	0.93	4.71
	Lung cancer (female)	0.38	0.90	3.80
	Breast cancer (female)	0.21	0.83	1.24
	Prostate cancer	0.24	0.78	1.09
	Cancer all (male)	0.34	0.85	2.27
	Cancer all (female)	0.34	0.85	2.27
Fixed	Lung cancer (male)	0.30	0.92	3.75
	Lung cancer (female)	0.33	0.92	4.13
	Breast cancer (female)	0.20	0.87	1.54
	Prostate cancer	0.17	0.78	0.77
	Cancer all (male)	0.30	0.91	3.33
	Cancer all (female)	0.26	0.89	2.36

The impact of applying different bandwidth sizes was assessed for ‘all cancers’ in men and women. The results of the overlay analyses were summarized in Additional file 1 and the sRRF maps are displayed in Additional files 2 and 3. Halving the bandwidths (S1), both risk estimators showed an increase in sensitivity, while the specificity, and thus the LR+ were increased only for the adaptive estimator. Halving the pilot and doubling the global bandwidths (S2) led to a similar sensitivity as in S1, but it reduced the specificity and thus lowered the LR+. By contrast, scenario 3 (S3) markedly reduced the sensitivity while raising the specificity; the LR+ was slightly lower than in S1. Doubling both bandwidths in the adaptive approach (S4) resulted in a sensitivity comparable to that in Table 2, but specificity and LR+ dropped to their lowest values. This contrasted with the fixed estimators, where despite a marked drop in sensitivity the increased specificity resulted in the highest LR+.

## Discussion

Existing studies that compare the fixed against the adaptive bandwidth-based estimation of the sRRF use either a simulation design, where the data arise from a standard parametric distribution with an a priori known risk surface [28,45], or real-world data, where the data rarely follow a standard distribution and the risk surface is commonly unknown [1,11,14,46,47]. The novelty of this study is that it combines the advantages of both study types: we use real-world data and a known risk surface to evaluate the performance of both bandwidth types in estimating the sRRF. We artificially created ‘risk areas’ for cancer by application of a spatial sampling method for the controls that is recognized as being biased to underestimating the population density in urban areas.

When employing in our study the default setting for automatic bandwidth selection – as implemented in *sparr* –, the optimal bandwidth was inversely related to sample size for both methods (OS principle and LSCV score). This is a known property of the optimal bandwidth [48]: the larger the sample size the less smoothing is required. The OS bandwidths were also consistently wider than for the LSCV score, because the OS method applies ordinary scale estimators such as the standard deviation or the interquartile range and these are inclined to place upper bounds on the smoothing parameter and hence produce conservative density estimates which tend to eliminate “accidental features” [37]. The differences in the two OS bandwidths (OS h(pooled) and OS* h(pooled) in Table 1) are caused by the different definitions of the overall sample size used in the denominator for calculating this bandwidth [28,37]. For the fixed risk estimator, the overall sample size (n_f_ + n_g_) is used as described by Terrell [38]. However, Davies and Hazelton [28] suggest to use $\sqrt{n_f*{n}_g}$ (=geometric mean) instead in an attempt to define an ‘effective’ sample size for the adaptive sRRF. Since ((n_f_ + n_g_) > ($\sqrt{n_f*{n}_g}\left)\right),$ the OS bandwidth for the fixed is smaller than that for the adaptive risk estimator. The LSCV bandwidth selector aims to find an optimal bandwidth in minimizing the mean integrated squared error (MISE) between the kernel estimator ($\widehat{f}$) and the true density (f) [37] which implies a known tendency of undersmoothing. It has also been recognized, that the LSCV score tends to show multiple local minima which are occasionally minimized at h = 0 [24,26,49]. This has been particularly observed when the sample sizes increase and clusters of observations at the same or nearly the same location are likely [48,50-52]. This could be one possible explanation for the “counterintuitive” observation of larger bandwidths for the spatial case patterns and may indicate a possible failure of the LSCV method to find a minimum bandwidth which minimizes the MISE.

The OS principle was elected due to its potential to control excess variability in the estimated densities [28]. However, if the risk is constant across the study area, the fixed estimator naturally overestimates the relative risk in rural areas due to a too small bandwidth. In turn, in urban regions, the risk is underestimated due to too large bandwidths relative to the underlying population density. In our study, the risk was assumed to be a function of the underlying biased population density, that is, the relative risk was artificially increased (due underestimation of the population density) in urban areas and decreased in rural areas. This explains much of the generally lower sensitivity of the fixed estimator: the main portion of the risk areas was located in urban areas where the fixed estimator has a priori a reduced sensibility to detect the risk areas. On the other hand, this led to reduced FPs and a higher LR+ as compared to the adaptive estimator. The adaptive estimator was introduced to reduce this bias by an adjustment of the bandwidth inversely to the underlying case resp. control density. Therefore, the adaptive risk estimator had a greater variability in urban areas, leading to more detected risk areas, but at the cost of a reduced specificity and therefore a lower LR+.

These patterns were most clearly observable in ‘all cancer’, because the case density reflects (more or less) the true population density: areas of high case densities were therefore strongly linked to areas of the most pronounced underestimation (due to the sampling with bias) of the control densities, thus producing the largest relative risk differences. When regarding the other cancer types, sensitivity and specificity were additionally influenced by cancer-specific processes, such as regional over- or underreporting or the presence or absence of screening bias. For example, the case density for ‘lung cancer’ was particularly high in the south of the study region, a district with large agglomerations of heavy industries and coal mines. Thus, the high lung cancer incidence attributable to smoking among the industrial workers may have been compounded by additional occupational and environmental exposures to high doses of dusts, foams and other industrial emissions. Therefore, the sensitivity of both estimators was fairly similar to that for ‘all cancers’ because the majority of risk areas are located in the south of the study area. But compared to ‘all cancer’ the specificity was also similar, because the fixed estimator detected an unusual large risk area north of the lung cancer agglomeration, probably due to a too small bandwidth in this rural setting (higher FP rate). This difference in specificity regarding the rural areas emerged more clearly in the ‘prostate cancer’ data. Here, the highest case density was found in the north, a less populated region in the study areas possibly related to overdetection of ‘prostate cancer’ by an active prostate cancer center which has a northern catchment area. The generally low sensitivity for this tumor type is attributable to the fact that only few risk areas were a priori located in this region due to an overestimation of the source population. While differences in sensitivity between the two estimators were largest, the specificity was exactly the same. And again too small bandwidths might have caused these enlarged risk areas as compared to the adaptive sRRF. Expectedly, the spatial distribution of ‘breast cancer’ was more concentrated in urban areas because participation in - at this time - an opportunistic screening (e.g. mammography offered at gynecologist contacts) was more prevalent here. On the other hand, several regions, in particular in the south of the study area, showed low screening participation rates which have been shown to be associated with reduced socioeconomic resources at individuals and regions level [53,54]. Indeed, the risk surface resembles that for ‘all cancer’ but with a reduced overall sensitivity due to the reduced case density. Here, again the concentration of the case density mostly over urban (‘risk’) areas explains the sensitivity and specificity pattern: The fixed estimator oversmoothed the relative risk in urban areas due to larger bandwidth, but produced simultaneously fewer FPs, resulting in an increased LR+.

In summary, we observed that the fixed risk estimator was characterized by a conservative behavior in the urban (‘risk’) areas i.e., less sensitive while avoiding FP alarms. In contrast, when the risk differences coincided with sparse data (= rural areas), especially seen for ‘prostate cancer’, the adaptive risk estimator profits from a variance stabilization due to a larger bandwidth, resulting in higher sensitivity and comparable specificity. These findings correspond well with the results of Davies and Hazelton [28]. Zhang et al. [14] applied the adaptive and fixed bandwidth-based sRRF to 80 cases and controls of schistosomiasis in the Guichi region (China) and found that the adaptive sRRF had a better ability to depict the spatial heterogeneity of sparsely populated risk areas.

Varying the originally derived bandwidth sizes for ‘all cancers’ accentuated the bias-variance tradeoff of the fixed sRRF: A reduction of the bandwidth (S1) reduced the difference between the density estimate and true density (bias), but at the expense of a higher variability of the estimate (variance). Therefore, a higher sensitivity was offset by a reduced specificity (mainly in rural areas). A doubling of the bandwidth (S4) led to the inverse process: the higher specificity due to a reduced variance of the risk estimate came at the cost of a higher bias (reduced sensitivity). Varying the adaptive bandwidths is more complicated, because at least two different bandwidths must be selected. Here, we observed that the global bandwidth affected the specificity/precision of the sRRF: when it was large (S2 &S4), specificity was a generally lower due to a reduced variability of the sRRF (oversmoothing) as compared to a smaller global bandwidth (S1 & S3). The pilot bandwidth determines the degree of preserved spatial heterogeneity present in the distribution of cases resp. controls, and it seems that this pilot bandwidth influenced the accuracy/sensitivity of the risk estimator. Therefore, halving both bandwidths led to an increase in sensitivity and specificity due to a more precise and accurate estimation of the densities, especially in the urban risk areas. In contrast, doubling the both bandwidths led to a loss of spatial precision due to oversmoothing, resulting in the lowest specificity.

Our results highlight the relative importance of choosing the optimal global bandwidth, because it essentially controls the specificity of the sRRF. Therefore, other bandwidth selection methods should be approved for selecting the global bandwidth. For instance, Davies [45] found promising advantage(s) in using the asymptotic mean integrated squared error (AMISE) criterion for selecting the global bandwidth. In another study, Davies [55] suggested to use a triangle dimension as scale estimator in the OS principle. However, these selectors have not yet been investigated for the adaptive sRRF. A further point to consider is the use of HR population grids for selecting a spatial sample of disease-free controls. This is a novel aspect that will become more popular with a better availability of an increased spatial resolution of these grids, because it is a time and cost effective way to obtain these controls. At present, freely available populations grids with a resolution of 100m exist for different countries of the world [56,57], but these grids are still too coarse to permit a regionalized monitoring of disease risks in small areas.

### Strengths and limitations

This study has several strengths and limitations. In the first place, the use of real world cancer data with a sufficiently large sample size as well as the use of a known risk surface, which in practice is always unknown, increased the precision and the credibility of the study results. A further advantage with regard to implementation aspects is the restriction to the exclusive use of open-source data and software environments given the often limited financial resources to conduct regional monitoring. Limitations arise from the fact that we explicitly searched only for elevated risk areas while there are also areas with decreased disease risks. However, generally the detection of risk areas is of greater public health interest than the identification of low risk areas or cold spots. Another limitation may have resulted from the assumption of a constant cancer risk across the underlying, but biased, population density, because additional processes may violate this lead to misclassifications of the sRRF. This should, however, have had no impact of the comparison of the two methods because the same sample of cases is used in both methods. Another critical aspect is the statistical testing for obtaining the p-values. Here, only the z-test statistic-based asymptotic normality test was used because these tests are computationally far less expensive, and appear more stable in sparsely populated areas than tests based on Monte-Carlo (MC) randomizations. However, given the large sample size we regard this source of bias as relatively small.

## Conclusion

In this study, we compared the fixed and the adaptive bandwidth-based sRRF in their ability to detect ‘risk areas’ for the occurrence of different cancer types. We observed that the fixed sRRF oversmoothes the risk in urban regions probably due to a too large bandwidth while the fixed estimator behaves conversely in rural settings. Because in our study design most risk areas were located in urban areas (due to a biased source population estimation), the fixed sRRF shows mostly a conservative behavior with reduced sensitivity but increased specificity, compared to the adaptive risk estimator that shows increased sensitivity in these urban areas at the cost of a lower specificity. Our results indicate that in situations where the risk was more concentrated in rural regions (e.g. due to sparse data) compared to the fixed sRRF, the adaptive risk estimator benefits from variance stabilization. Furthermore, the automatically selected bandwidth sizes used for the adaptive sRRF appear only suboptimal, which highlights the need for optimizing existing, and investigating new bandwidth selection methods, in particular for global bandwidth.

## Additional files

Additional file 1:
**Summary of the spatial overlay analysis of the ‘risk areas’ with the significant p-contours (α=.05) of ‘cancer all’ using different bandwidths.** Sensitivity, specificity, and the positive likelihood ratio (LR+) are presented as area ratios. The different bandwidth sizes according to scenario S1 to S4 are given in the method section. Additional file 2:
**Sensitivity analyses using different bandwidths for ‘cancer all’ (male population).** Different scenarios (S1-S4) were applied for the adaptive (_adapt) and fixed (_fix) spatial relative risk estimators. The 5% significant tolerance contours are overlaid as solid black lines and the true risk areas as dotted purple lines. Additional file 3:
**Sensitivity analyses using different bandwidths for ‘cancer all’ (female population).** Different scenarios (S1-S4) were applied for the adaptive (_adapt) and fixed (_fix) spatial relative risk estimators. The 5% significant tolerance contours are overlaid as solid black lines and the true risk areas as dotted purple lines.
