# Supplementary material for: Comparing adaptive and fixed bandwidth-based kernel density estimates in spatial cancer epidemiology
Source: Int J Health Geogr. 2015 Mar 31;14:15. doi: 10.1186/s12942-015-0005-9 (PMC4389444; doi:10.1186/s12942-015-0005-9)
Supplement: Additional file 1: — Summary of the spatial overlay analysis of the ‘risk areas’ with the significant p-contours (α=.05) of ‘cancer all’ using different bandwidths. Sensitivity, specificity, and the positive likelihood ratio (LR+) are presented as area ratios. The different bandwidth sizes according to scenario S1 to S4 are given in the method section. [file 12942_2015_5_MOESM1_ESM.pdf]

Additional file 1: Summary of the spatial overlay analysis of the 'risk areas' with the significant p-contours (α=.05) of 'cancer all' using different bandwidths. Sensitivity, specificity, and the positive likelihood ratio (LR+) are presented as area ratios. The different bandwidth sizes according to scenario S1 to S4 are given in the method section.

Bandwidth	Cancer type		Sensitivity	Specificity	LR+
Adaptive	Cancer all (male)	S1	0.41	0.91	4.56
	Cancer all (female)	S1	0.40	0.91	4.44
	Cancer all (male)	S2	0.37	0.80	1.85
	Cancer all (female)	S2	0.37	0.80	1.85
	Cancer all (male)	S3	0.26	0.93	3.71
	Cancer all (female)	S3	0.25	0.93	3.57
	Cancer all (male)	S4	0.37	0.74	1.42
	Cancer all (female)	S4	0.33	0.77	1.43
Fixed	Cancer all (male)	S1	0.44	0.85	2.94
	Cancer all (female)	S1	0.38	0.87	2.92
	Cancer all (male)	S4	0.25	0.95	5.00
	Cancer all (female)	S4	0.25	0.95	5.00
