# Supplementary figures and images for: Comparing adaptive and fixed bandwidth-based kernel density estimates in spatial cancer epidemiology
Source: Int J Health Geogr. 2015 Mar 31;14:15. doi: 10.1186/s12942-015-0005-9 (PMC4389444; doi:10.1186/s12942-015-0005-9)

North

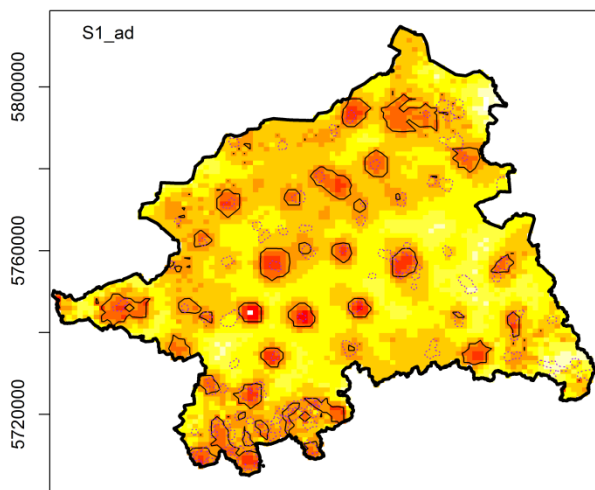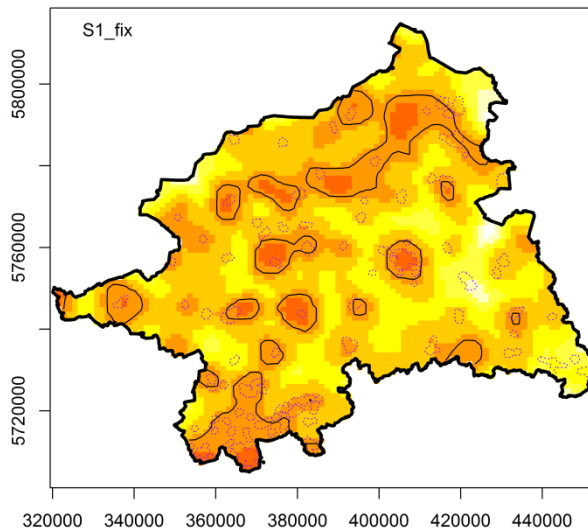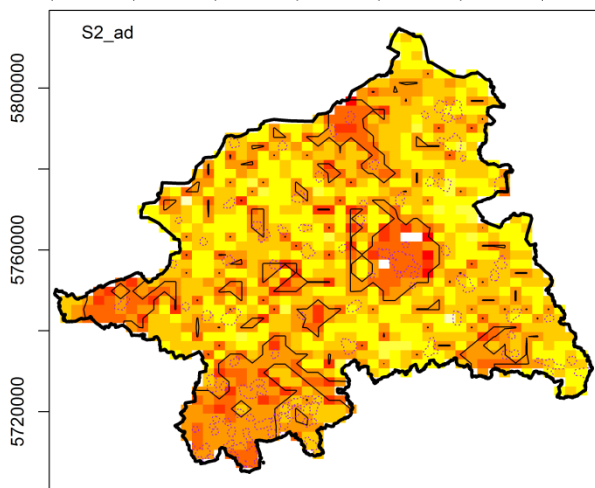

Log RR

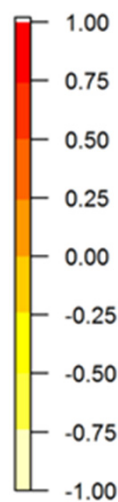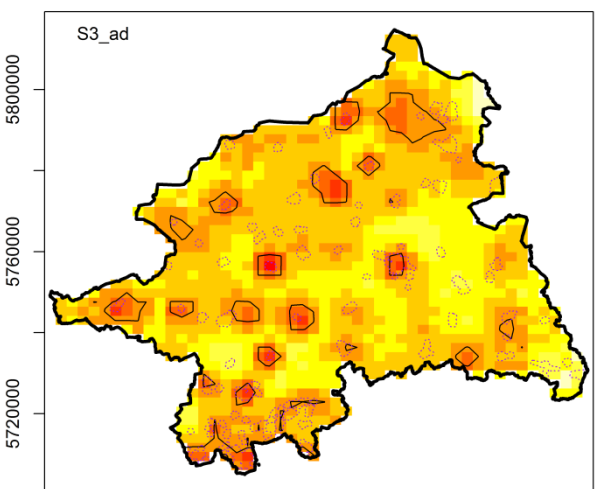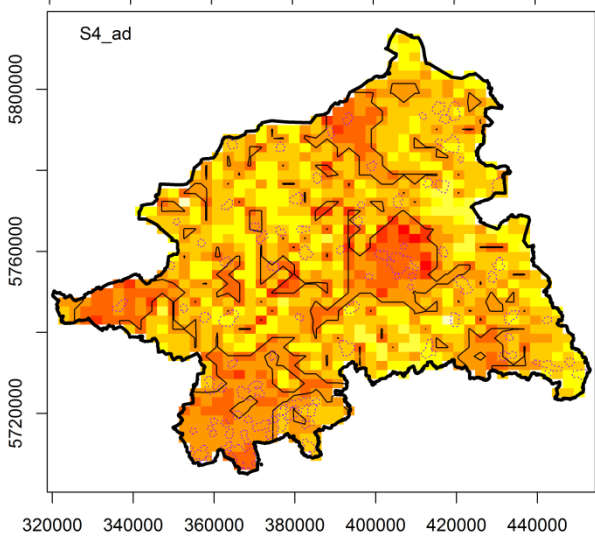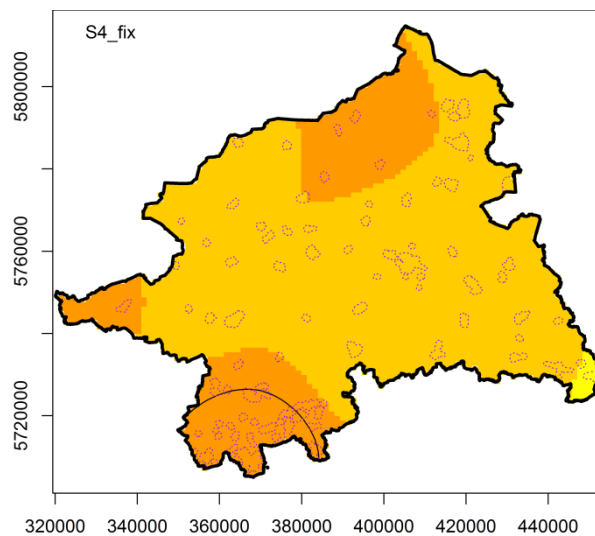

East

Supplement: Additional file 2: — Sensitivity analyses using different bandwidths for ‘cancer all’ (male population). Different scenarios (S1-S4) were applied for the adaptive (_adapt) and fixed (_fix) spatial relative risk estimators. The 5% significant tolerance contours are overlaid as solid black lines and the true risk areas as dotted purple lines. [file 12942_2015_5_MOESM2_ESM.pdf]

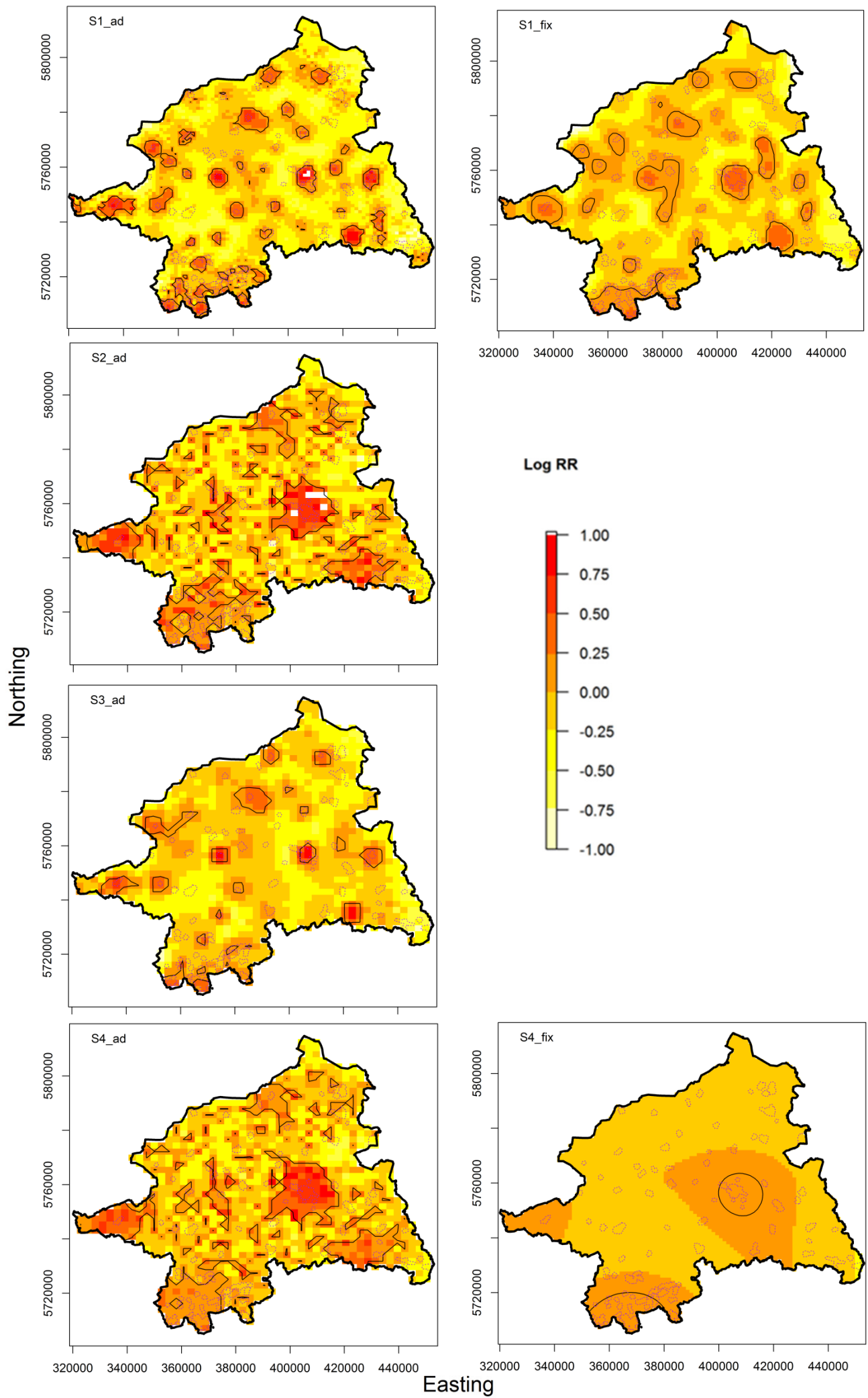

Supplement: Additional file 3: — Sensitivity analyses using different bandwidths for ‘cancer all’ (female population). Different scenarios (S1-S4) were applied for the adaptive (_adapt) and fixed (_fix) spatial relative risk estimators. The 5% significant tolerance contours are overlaid as solid black lines and the true risk areas as dotted purple lines. [file 12942_2015_5_MOESM3_ESM.pdf]
